# HMMER web server: 2015 update

**DOI:** 10.1093/nar/gkv397

**Published:** 2015-05-05

**Authors:** Robert D. Finn, Jody Clements, William Arndt, Benjamin L. Miller, Travis J. Wheeler, Fabian Schreiber, Alex Bateman, Sean R. Eddy

**Affiliations:** 1 European Molecular Biology Laboratory, European Bioinformatics Institute (EMBL-EBI), Wellcome Trust Genome Campus, Hinxton, Cambridge, CB10 1SD, UK; 2 HHMI Janelia Research Campus, 19700 Helix Drive, Ashburn, VA 20147, USA; 3 Department of Computer Science, University of Montana, Social Sciences Building Room 412, Missoula MT 59812, USA

## Abstract

The HMMER website, available at http://www.ebi.ac.uk/Tools/hmmer/, provides access to the protein homology search algorithms found in the HMMER software suite. Since the first release of the website in 2011, the search repertoire has been expanded to include the iterative search algorithm, *jackhmmer*. The continued growth of the target sequence databases means that traditional tabular representations of significant sequence hits can be overwhelming to the user. Consequently, additional ways of presenting homology search results have been developed, allowing them to be summarised according to taxonomic distribution or domain architecture. The taxonomy and domain architecture representations can be used in combination to filter the results according to the needs of a user. Searches can also be restricted prior to submission using a new taxonomic filter, which not only ensures that the results are specific to the requested taxonomic group, but also improves search performance. The repertoire of profile hidden Markov model libraries, which are used for annotation of query sequences with protein families and domains, has been expanded to include the libraries from CATH-Gene3D, PIRSF, Superfamily and TIGRFAMs. Finally, we discuss the relocation of the HMMER webserver to the European Bioinformatics Institute and the potential impact that this will have.

## INTRODUCTION

Homology searches are widely used within molecular biology, facilitating the transfer of annotation from a functionally characterised sequence or region to a corresponding region in another sequence. When searching against sequence databases, the HMMER software uses profile hidden Markov models (HMMs) to represent the query (1,2)—which can take the form of a single protein sequence or a multiple sequence alignment. In the case of a multiple sequence alignment, the observed amino acid frequencies in each column are converted to position-specific probabilities, with per position probabilities for both insertions and deletions, determined from the input alignment (1,2). For single sequence searches, a profile HMM is constructed from the sequence using position-independent affine gap open and extension probabilities (defaults: 0.02 and 0.4) and emission probabilities obtained from the inferred probabilistic basis of a standard substitution matrix (default: BLOSUM62) (3,4).

In early 2011, the functionality of the website hosting the HMMER software (http://hmmer.org) was expanded to allow online searches of protein sequences against either a protein sequence database or a HMM library (5). This search service not only took advantage of the speed improvements of HMMER3 software (6), but also hardware, the latest approaches to website design and other technical implementations (e.g. in-memory databases and use of NoSQL). The combination of these four aspects allowed the searching of sequences against large sequence databases such as UniProtKB, at near interactive speeds. Websites with minimal loading times (<10 s) are recognised for not interrupting the user's train of thought, and hence increase user productivity (7–9).

Since the initial release, the popularity of online HMMER searches has grown, with millions of sequence searches performed per year (averaging over 5200 searches per day or one search every 6 s, search statistics for http://hmmer.org only). These searches are split between requests coming from a browser (20% of searches) or via programmatic access using the RESTful application program interface (API, 80% of searches). For example, RSCB-PDB (10) uses the API to annotate newly deposited structures with Pfam annotations.

The initial release of the website provided the following three search algorithms:

- *phmmer*—single protein sequence against protein sequence database
- *hmmscan*—single protein sequence against profile HMM library (Pfam)
- *hmmsearch*—either multiple sequence alignment or profile HMM against protein sequence database

In this article, we describe the recent developments of the website, which include iterative searches with *jackhmmer -* an expanded repertoire of HMM libraries and a variety of result visualisations that allow rapid interrogation and interpretation of the results. Such developments are set against a constant background of target database growth, which for the large sequence databases results in a constant increase in the dynamic range of hits that will be returned from a query. We demonstrate different ways that searches can be limited and how the results can be progressively dissected according to taxonomy and/or domain architecture (the order of the domain(s) on a sequence).

## HMMER WEBSERVER DEVELOPMENTS

The HMMER webserver has been upgraded to the latest version of the HMMER software, version 3.1b2. This software version includes minor bug fixes, but more importantly has additional performance improvements over the previous version, version 3.0. While this has made the searches faster, the underlying sequence databases have grown substantially. UniProtKB (11) currently contains 91 408 504 sequences (release 2015_02), compared with 13 593 921 in January 2011 (release 2011_01), an increase of 570%. One of the major speed optimisations utilised by the site requires the caching of the sequence database in memory. Due to exponential growth of the databases, it is no longer possible to support both the NCBI non-redundant protein sequence database and UniProtKB. Future software developments will ultimately allow the sharding of the databases to allow future scalability, but this has yet to be implemented within the HMMER daemon (*hmmpgmd*). Consequently, the website now focuses primarily on UniProtKB (11) as it represents the world's pre-eminent protein database, with sequences annotated either by expert curation or by the application of expert curated rules for automated annotation. While searches remain fast, returning in a matter of a few seconds, subsets of UniProtKB have been included to provide either the highest quality (UniProtKB/Swiss-Prot) annotations, representative sets to provide good coverage of sequence space while reducing the number of potential matches (UniProt Reference Proteomes (11) and Representative Proteome sets (12)) or for curation purposes (pfamseq—Pfam's underlying sequence database). While these subsets do not change the amount of data cached in memory, the smaller target databases increase search performance and make results more manageable. We also include sequences from known structures that have been deposited in the Protein Data Bank (13)).

The growth of UniProtKB has been substantial, but it is important to remember that increasing fractions of the new sequences are either identical or nearly identical (>95% identity) to a sequence that already exists in the database (11). Given the nature of this sequence database growth, it is unsurprising that the number of sequences that a query may match from a homology search has equally grown. Thus, many of the developments of the website have focused on trying to improve results visualisation, from summaries to alternative representations to filtering.

### Expanded results visualisations

User experience testing and web usage statistics indicate that it is very difficult to predict what a user is trying to achieve from a homology search. A user's purpose for the search can range from functional annotation to establishing taxonomy distribution to understanding residue conservation in a collection of aligned sequences. The developments described in the following sections have all been designed to enhance access and understanding of the results, whilst catering to a wide range of use cases or enquiries.

#### Sequence matches and features

**Figure 1.**
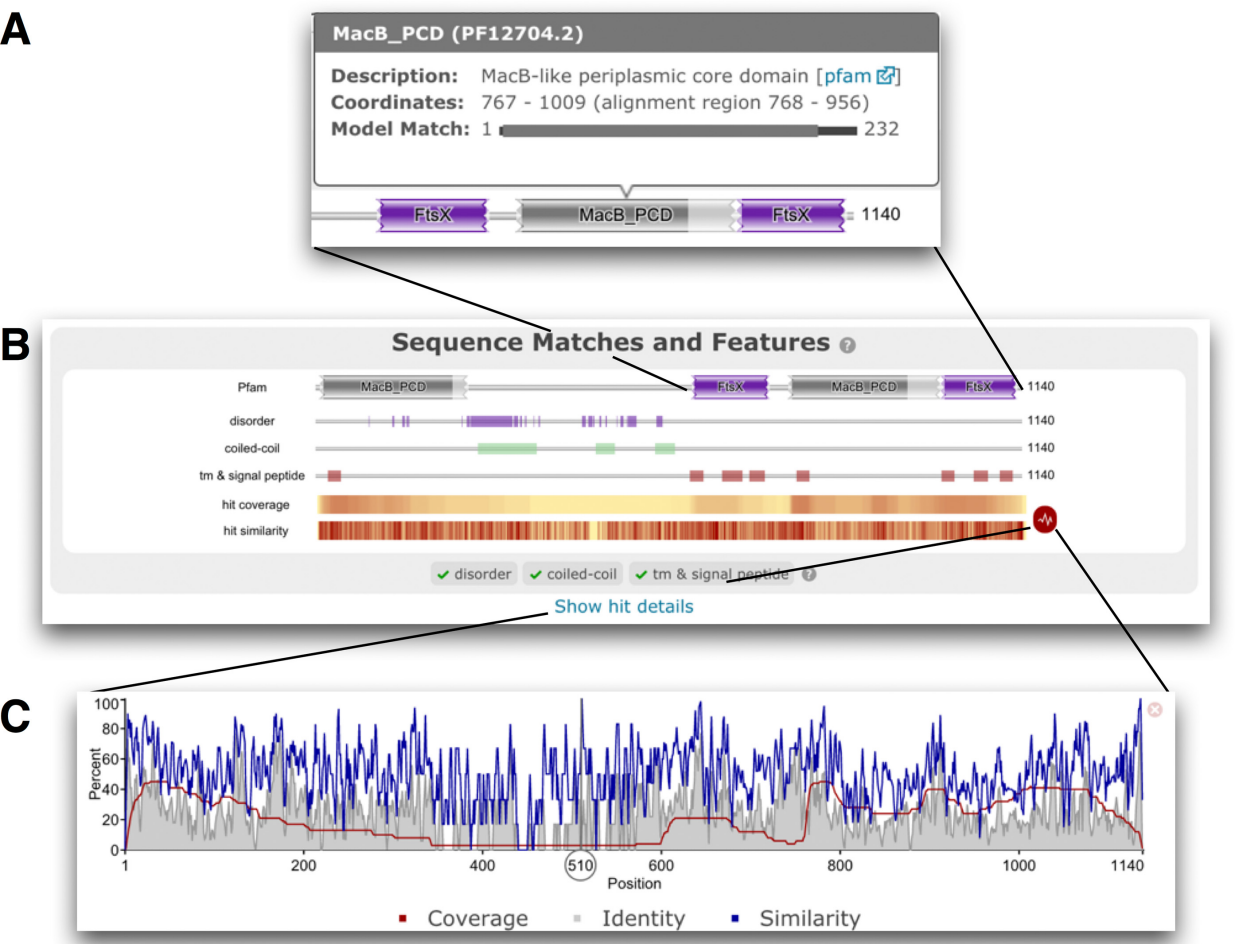
Results of searching the Efflux ABC transporter permease protein from *Enterococcus casseliflavus ATCC 12755* (UniProtKB accession F0EMD7) against the Reference Proteome database using *phmmer* with default search options. (**A**) The tool tip associated with the partial C-terminal MacB_PCD domain match. The model match line indicates the region of the HMM to which the sequence has been aligned (alignment region). While the match is incomplete, in this particular case, >90% of the model positions have been matched. (**B**) Shows the Pfam matches on the query and other sequence features. The hit coverage and similarity are shown in a condensed heat map style view below the sequence features. These can be expanded using the red icon to their right. (**C**) The hit similarity and coverage graph, summarising the *phmmer* matches.

When performing a *phmmer* search, the single sequence query is also automatically searched against the Pfam (14) profile HMM library using *hmmscan*, to identify the presence of any Pfam families on the query sequence. The result of the Pfam search is displayed at the top of the page, within the ‘Sequence Matches and Features’ section. The graphical representation of Pfam domains remains unchanged—for Pfam entries that are considered type ‘domain’ or ‘family’, a rectangular shape with curved ends represents a full-length match position graphically. However, when a hit does not match the first and/or last state of a profile HMM, a jagged end represents the N-terminal and/or C-terminal end of the match. While it is informative to know that a match is not full-length, it does not provide the user with the concept of how incomplete the match is compared to the profile HMM. As HMMER uses a local-local match strategy (a match can be anywhere in the sequence or the HMM) partial matches are common. For all Pfam matches, information on the completeness of match is now provided in the tool-tip (revealed by placing the mouse cursor over the graphical representation of the domain), where the profile HMM is represented by a black bar and the region matched in the profile HMM indicated by an overlaid coloured rectangle. As shown in Figure 1A, this gives an immediate impression of length of the match between the sequence and the profile HMM, even when it is not full length.

The original ‘Sequence Matches and Features’ view has been advanced further to include other types of annotation and to provide a summary of the *phmmer* search results (Figure 1B, C). The protein sequence is also now analysed for the presence of other features: disordered regions using IUPred (15,16), signal peptides and transmembrane regions using Phobius (17) and coiled-coil regions (18). When a sequence contains one or more matches against one of these three algorithms, a graphical representation showing the positional information from each algorithm is dynamically inserted under the Pfam domain graphic. If a sequence does not contain any matches, a graphic is not displayed. However, the successful execution of the different feature algorithms is shown below, within the bottom border of the bounding box (green check mark on success, red x mark on failure). Figure 1 shows an example of the interplay between these annotation tools. In this example, there is a large region of sequence between the N-terminal MacB_PCD (Pfam accession:PF12704) and the FtsX domains (Pfam accession:PF02687) that is not currently represented by a Pfam domain. Inspection of these other sequence features indicates that this is a region that is expected to be largely disordered and contains three coiled-coil motifs. While neither feature type precludes a Pfam entry from being present, such features are typically less tractable as they are often poorly conserved at the amino acid level.

Upon completion of the associated *phmmer* search, all of the matches are aligned and used to display two additional tracks in the ‘Sequence Matches and Features’ section, the hit coverage and the hit similarity. Both tracks use a heat map style to represent the two hit metrics. The hit coverage indicates the regions of the query sequence that have been matched by the sequences in the target database. As matches can be anywhere between the query and target, the presence of a ubiquitous domain or motif in the query can result in many sequences matching the query that may be overall functionally distinct yet share the common homologous domain. Figure 1B shows an example of how the hit distribution varies across the sequence, from pale yellow (little coverage) to red where there is more coverage. The second track below shows the relative sequence similarity of the sequences aligned at each position. This track clearly indicates that the sequence similarity fluctuates across the sequence, but patches of high similarity can be identified that align with the transmembrane regions in the C-terminus of the sequence. A more detailed view of the information contained in both tracks can be obtained by clicking on the icon to their right. This reveals a graph that plots the relative hit similarity, relative hit identity and the percentage occupancy of the column in the alignment (match positions). Moving the mouse cursor over the graph reveals a moving line, which allows the position of the graph/alignment to be more readily determined. Overall, these additional developments allow a rapid understanding of the domains, sequence features and conservation profile of the hits found in the *phmmer* search.

### Viewing results in different formats

When performing any search against a sequence database (*i.e. phmmer, hmmsearch* or *jackhmmer*), the default result view is a paginated tabular scores output, with matching target sequences ranked according to bit score (high to low), which corresponds to an ordering by expectation value (E-value, ordered low to high, most significant to least). The histogram above the results table, termed ‘Hit graph’, summarises the distribution of hits according to both E-value and taxonomy. The *x*-axis of the histogram is divided into 30 E-value bins, ordered from least to most significant, with the total height of each column (or bin) proportional to the number of hits that fall within the bin. Each column in the histogram is further subdivided according to the major taxonomic group, with the size of the bar proportional to the number of hits in that group. Clicking on columns in the histogram takes the user to the row in the results table of the most significant hit in the bin represented by that column.

**Figure 2.**
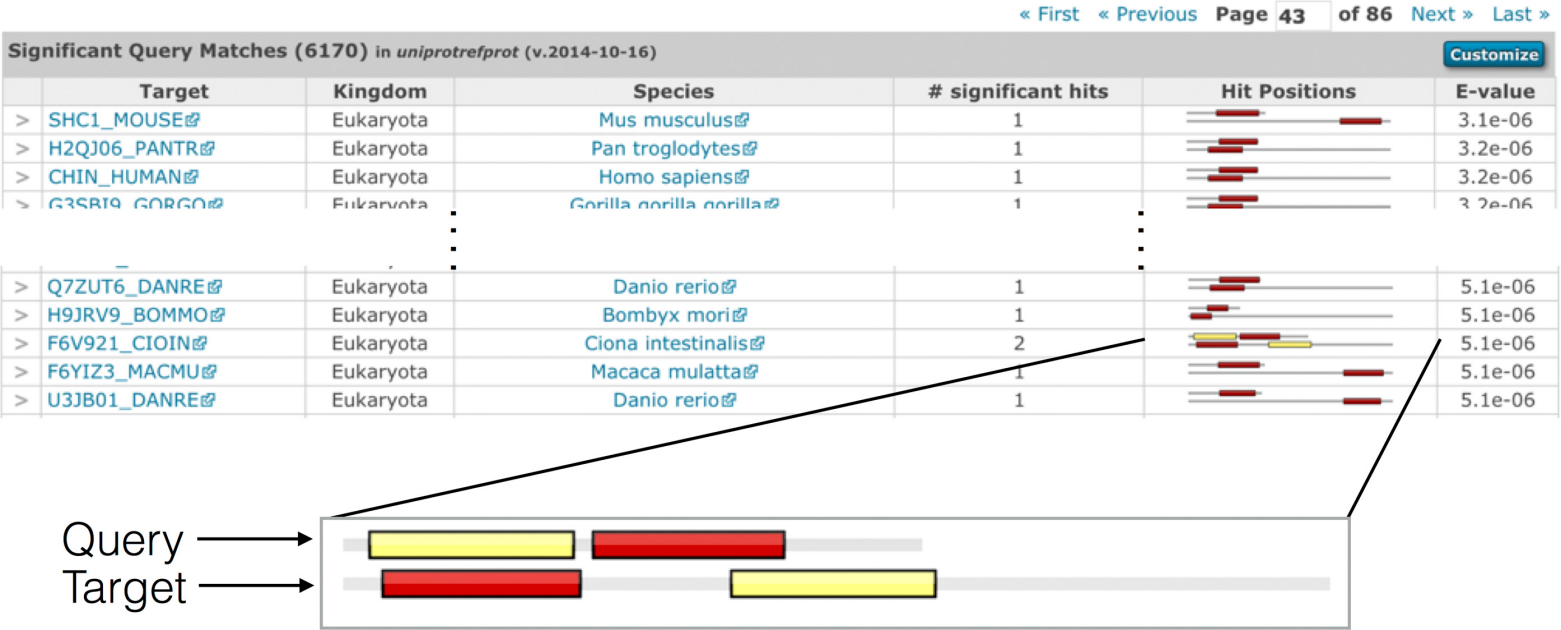
Example of the expanded results table, showing the kingdom and species, number of significant hits, and the hit positions between the query and the target sequences after searching the UniProtKB sequence accession P00519 (amino acids 57 to 218) against the UniProtKB reference proteomes sequences (2014_10 release). The customise button in the top-right of the table header can be used to switch on different columns in that table (row count, secondary accessions, description, species, kingdom, known structure, number of identical sequences, number of hits, number of significant hits, bit score and graphical representation of the hit position). An expanded view of the hit position graphic is shown below the table. The enlarged view indicates where the two regions of similarity, or hits, in the query sequence match the target sequence. Each distinct hit of the query sequence is shown as a coloured box, and the corresponding aligned region is represented by a box of the same colour. The two sequences in each row are drawn proportionally to each other, with the sequence represented as a grey line. The two sequences are drawn left-justified (i.e. unaligned), with the query sequence always shown above the target. In this particular case, the order of the hits is reversed between the query and target sequences. A similar representation is used for queries with a profile HMM, with the top image (the query) representing the length of the profile HMM. The hit graphic quickly allows the identification of sequence rearrangements and repeated regions (where hit/coloured box in the query is duplicated multiple times in the target sequence).

The results table is now customisable to allow the user to include a range of additional data fields that provide information on the hit sequences and nature of the match. Figure 2 shows an example of the ‘Score’ results table, where these three additional columns (taxonomic classification of the organism to which each matched sequence belongs, number of significant hits, and a graphical display of the position of the hit(s) between the query and target) have been added using the ‘Customize’ button found in the header of the table. The highlighted example in Figure 2 illustrates the two hit regions between the query and target demonstrating that there has been a re-arrangement of the hit regions in the query sequence compared to the target sequence.

While the score view is a more typical way of viewing results, we have developed two alternative ways of visualising the results, (1) by taxonomy and (2) by domain. These views apply to the results of *phmmer, hmmsearch* and *jackhmmer* searches.

### Taxonomy view

**Figure 3.**
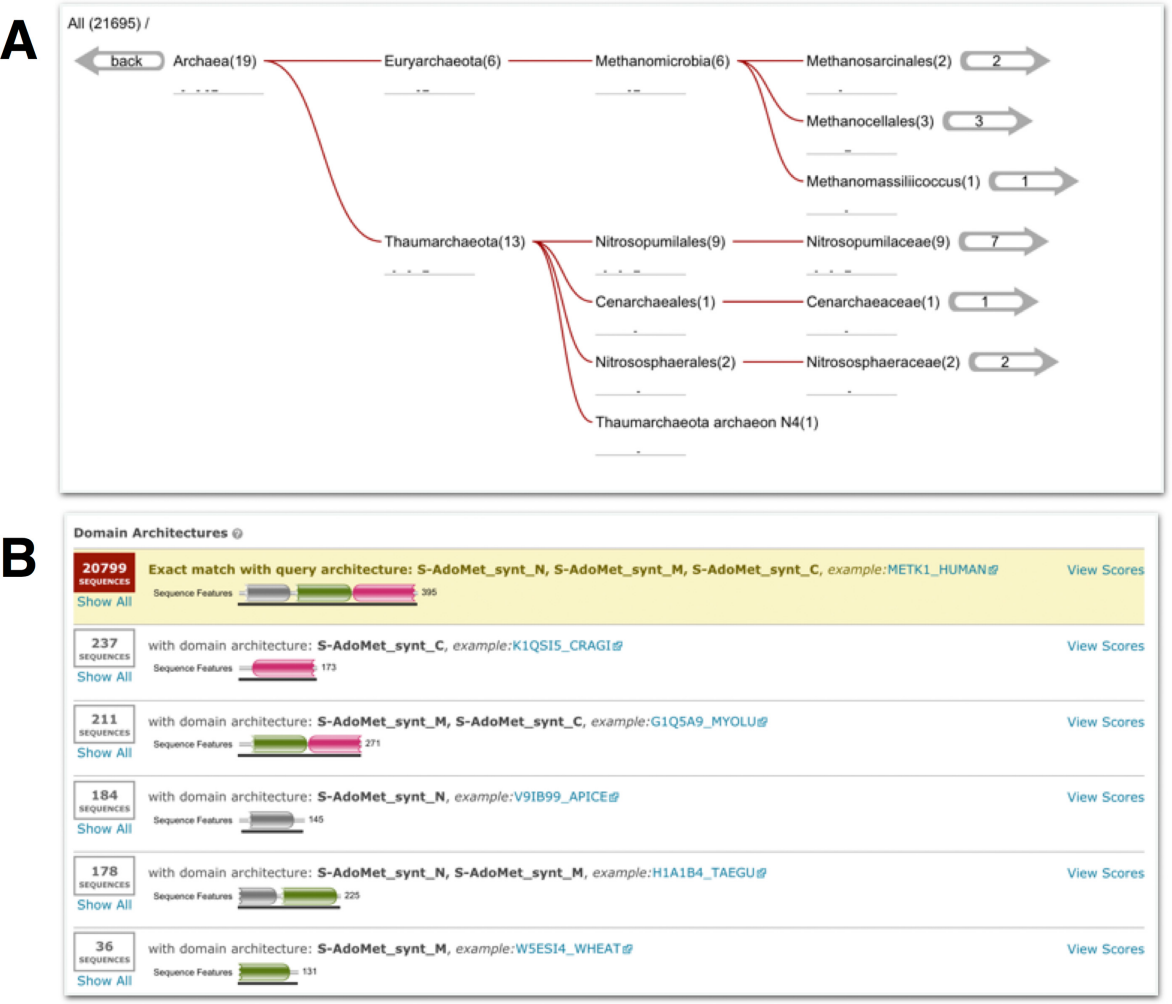
Two different results view from searching the human S-adenosylmethionine synthase sequence (UniProtKB accession Q00266) against UniProtKB (2014_10 release). (**A**) The taxonomic distribution of the archaeal homologs in the results. Below each taxonomic name is a sparkline version of the hit graphic showing the hit distribution of all sequences belonging to that taxonomic clade. The numbers in brackets denote the number of sequences matched, while the numbers in the right-hand arrows indicate the number of species. (**B**) The same results as in (A), but grouped according to domain architecture. In this example, 20 799 out of the 21 695 match sequences have the same domain architecture as the query (as indicated by the yellow background). The remaining domain architectures appear to be subsets of the dominant domain architecture, arising from sequence fragments found in the database.

The ‘Taxonomy’ view, Figure 3A, shows the taxonomic distribution of matches according to a species tree. The species tree is derived from the NCBI taxonomy (19) and drawn from left (root) to right. More often than not, the taxonomic distribution of the matches is too broad to display the entire taxonomic tree. By default, the tree is shown with the top four taxonomic levels found, but the user can click on the tree to focus on a specific lineage, allowing them to browse the most relevant clades and organisms while temporarily hiding other parts of the tree (Figure 3A). Each node in the displayed tree corresponds to a taxonomic level and shows a sparkline version of the ‘hit graph’ for that level to indicate the number of hits and their E-value distribution for that particular taxonomic level. The arrow(s) on the right side of the tree indicate the number of species that match below that level. Clicking on any of the nodes of the tree will re-focus the tree, such that this node appears on the left side of the tree representation and children nodes (names) are shown to the right (as many as exist, or are permitted by available space). Once the root of the taxonomic tree (*All*) is no longer visible, a breadcrumb trail of the viewed branch back to the root of the taxonomic tree is displayed above the tree. Either the breadcrumb trail or the back arrow on the left can be used to move back up the taxonomic tree (Figure 3A). In addition to changing the graphical tree, re-focusing the tree to different nodes causes the species listed in the table below the tree to be updated to show just the species and the number of hits for the taxa found below the visible root.

### Domain architecture view

A typical homology search against a large sequence database will return hundreds to thousands of hits. An alternative to clustering hits by taxonomy is to cluster them by the domain architecture of the hit sequences (the ordered collection of domain(s) found across the entire sequence). The ‘Domain’ view lists all of the unique domain architectures found in the set of matched sequences, with the domain architectures defined according to Pfam domains. All hit sequences containing exactly the same domain architecture grouped into a single row of the table. The number of sequences containing a given architecture is indicated on the left, and the scores of just this set of sequences can be viewed using the link on the right of the row. The table is ordered according to the frequency that each domain architecture occurs in the result set. If the query is a single sequence, then the row containing the same architecture as the query is highlighted (Figure 3B). Typically, this view of the results can represent over 75% of all the results in the first page, providing a rapid understanding of domain diversity of the matched sequences.

### Filtering search results using different views

**Figure 4.**
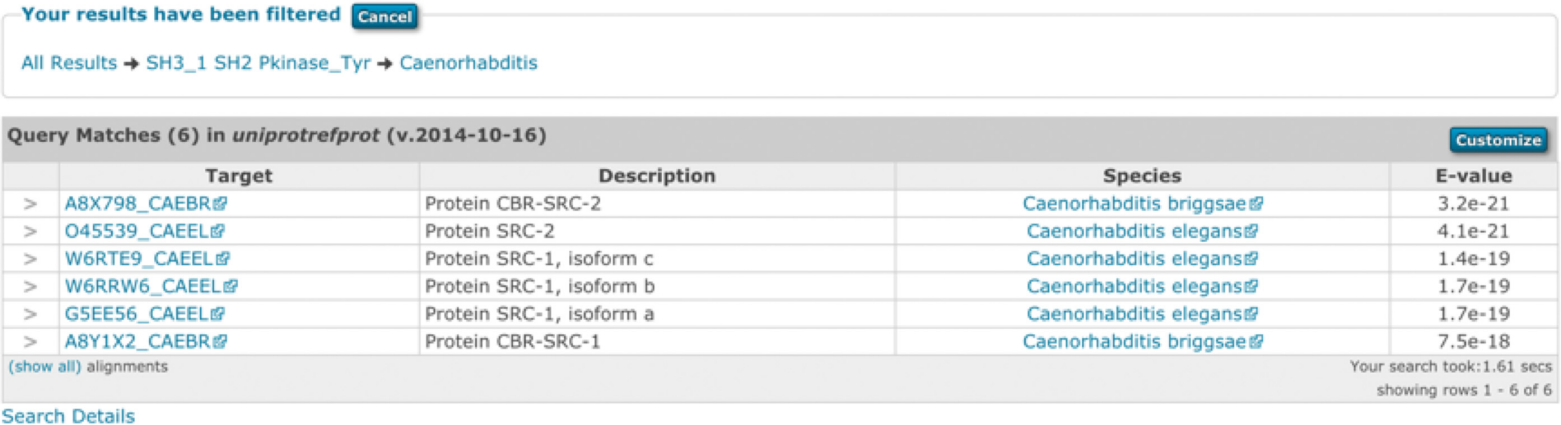
An example of filtered search results using both domain architecture and taxonomic filters (described in the text). The box above the table shows the filtering steps, first restricting by the domain architecture ‘SH3_1 SH2 Pkinase_Tyr’ then by a taxonomy filter. The user can click the filter labels in the breadcrumb string (‘All Results') in the filter section to reverse any of the steps to the right, or all filters can be cancelled by clicking the cancel button.

With the number of sequences deposited in the UniProtKB databases growing at unprecedented speed, an average query sequence might return an overwhelming number of hits. Classically, hits have been presented as raw tables, but in doing this it can be hard to find the most informative matches buried deep in the results. While using either taxonomy or domain architecture to provide alternative views of the data, ordering results by E-values remains an important way to prioritise matches. Consequently, the results interface has been developed so that both the domain architecture and taxonomy views can be used to filter the results, e.g. select hits belonging to a taxonomic clade and subsequently filtering the subset by domain architecture or *vice versa*. For example, using the default example query for a *phmmer* search, a user may wish to identify all *Caenorhabditis* sequences that contain the domain architecture ‘SH3_1’, ‘SH2’ followed by ‘Pkinase_Tyr’. To do this they would select the ‘View scores’ for this architecture, then select the taxonomy view and navigate to *Caenorhabditis* and select the ‘Show scores for all’, which reveals the 6 matching sequences from the 6170 matches, in a few simple clicks (Figure 4).

### Taxonomy-restricted searches

While the previous section describes filtering of results once they have been calculated, an alternative way of restricting the results it to reduce the initial search space. While alternative target databases offer one such mechanism, another approach (which can be used in combination with any of the sequence databases) is to restrict the search to sets of sequences belonging to one or more taxonomic clades using the ‘Restrict by taxonomy’ on the search submission page. This can be performed by either entering valid taxonomic levels (species, phylum) or checking taxonomic levels in a representative taxonomy tree provided on the website. Note that when entering different taxonomic terms, the look-up tool is aware of the taxonomic tree. For example, if a user wants to search all sequences from Chordates except human, they would not want to have to select species individually. To enable the rapid construction of such queries, the user would first enter *‘Chordata’* followed by ‘*Homo sapiens*’. As the first term has already selected ‘*Homo sapiens*’ (as it is part of ‘*Chordata*’), the query builder assumes that user wants to remove ‘*Homo sapiens*’ from the set of sequences to search. As taxonomic terms are added to the query, the interpretation of the terms by the query builder is displayed below the input field. Results from taxonomically restricted searches will be presented as described above, with the same score, taxonomy and domain architecture views, while also clearly indicating that the search space has been restricted. It is important to note that the E-values are calculated as if the entire target sequence database had been searched. Using such restrictions on the search improves search speeds, as well as improving result visualisation by focusing matches on the desired taxonomic range.

### Multiple HMM databases

The *hmmscan* algorithm takes a single protein sequence and searches it against a profile HMM library. The first profile HMM database to be incorporated into the site was the Pfam library. This initial display has been expanded to provide the disorder, coiled-coil, signal peptide and transmembrane annotations described earlier in the article. Furthermore, the HMMER3 based protein family databases CATH-Gene3D (20), PIRSF (21), Superfamily (22) and TIGRFAMs (23) have been incorporated into the *hmmscan* search as alternative target HMM databases. While Pfam and TIGRFAMs use the domain boundaries assigned by HMMER directly, CATH-Gene3D, PIRSF and Superfamily employ alternative post-processing methods for domain assignments. This is primarily because a family/domain may be represented by more than one profile HMM, or may have to reach additional criteria specified by the database e.g. length, and in the case of structural domains, the domain may not be contiguous on a protein sequence. Consequently, the standard *hmmscan* thresholds are disabled for these three databases and the significance thresholds/criteria provided by each database are applied. The E-value or bit score threshold can still be defined for either Pfam or TIGRFAMs.

Unlike in the selection of target sequence databases, it is now possible for more than one profile HMM database to be selected, allowing the different protein family assignments to be compared in a single search submission. As each annotation returns, it is inserted into the results page and shown both graphically and as a table where appropriate. If no matches are found for a particular protein family database this will be indicated in the list of tables below the graphical summary.

### Iterative searching

**Figure 5.**
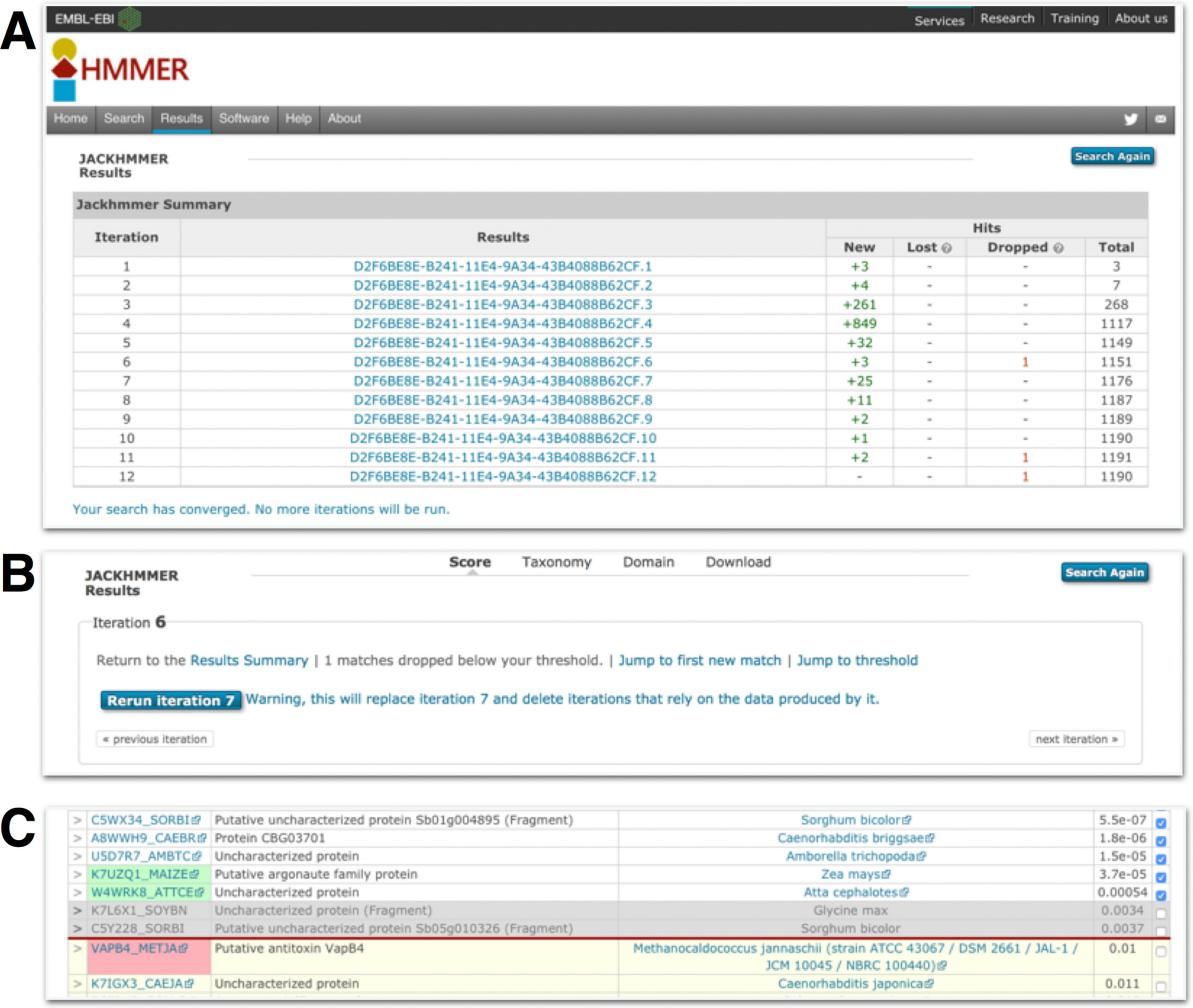
Examples of the *jackhmmer* user interface. (**A**) This shows the summary table of a *jackhmmer* search that has been iterated to convergence. Each iteration is compared to the previous stage and shows the number of new sequences found compared to the previous iteration, the number of sequences lost (see text for details), the number of sequences that were dropped and the total number of sequences. The results job identifier in the second column provides a link through to the results table for that iteration. At the top of the results page for a specific iteration, there is an ‘iteration’ box (**B**). This provides information about the iteration and a series of links to navigate to the summary page, or previous or next iteration results, to re-run iterations or to navigate the results. If any sequences have been lost, a link to a table listing those sequences is provided. (**C**) Shows the results on either side of the inclusion threshold (red horizontal line). The rows containing sequence accessions with a green background indicate new sequences that were not previously above threshold. The row containing a sequence accession with a pink background is a sequence that is no longer significant, but was in the previous iteration, i.e. dropped. The grey rows indicate the sequences that have been manually de-selected by the user and will not be used in the subsequent iteration.

The initial release of the HMMER website included the search algorithms *phmmer, hmmsearch* and *hmmscan*. The HMMER software package also includes *jackhmmer*, which on the command line allows a single sequence to be searched iteratively against a target sequence database, similar to PSI-BLAST (24) functionality. Iterative sequence searching is often able to identify similarities to functionally characterised proteins that are not detected with single sequence searching (25), as the residue conservation from a set of related sequences is used to determine position specific amino acid, insert and delete probabilities. This iterative search functionality has now been implemented in the HMMER website, but, unlike the command line version of *jackhmmer* which only accepts a single protein sequence as a query, the website implementation allows *jackhammer* to be initiated with a single sequence, a profile HMM or a multiple sequence alignment against a target sequence database (as with *phmmer*). When starting with a single sequence, the first round of *jackhmmer* is equivalent to *phmmer*; otherwise it is equivalent to *hmmsearch*, with the first results page reflecting the nature of the search method. In the case of a single sequence, the result page is shown with the ‘Sequence Features and Matches’ information. A further notable difference between the command line version of *jackhmmer* and the website implementation is that the website allows the user to interact with the results from one search iteration before starting the next, by either including or excluding sequences (Figure 5A, B, C). Under the menu for the different result visualisations (Figure 5B), an ‘iteration count’ box indicates the current iteration and contains links that allow the user to either jump to the hit at the inclusion threshold of the search, or show the results summary of the different iterations (2 or more iterations) and start the next iteration. In the scores table, the user can use the check boxes (located in the right most column, by default all hits above the threshold are preselected) to either include or exclude sequences from the search results; a checked box indicates that a sequence will be included in the next round, even if it currently falls below threshold from the preceding search. When a sequence is removed that is currently scoring above threshold, the row will be shown in grey (Figure 5C).

After the first iteration, regardless of the initial input, the HMMER web server builds a profile HMM from the selected sequences and searches it against the sequence database, equivalent to an *hmmsearch* search. Rather than immediately going to the results page, a *jackhmmer* summary table is presented to the user, comparing the results of each iteration to the previous round (Figure 5A). The table lists the iteration, links to the results and lists the number of new sequences found, the number lost, the number dropped and the total number of sequence matched in that round. Sequences that have been ‘dropped’ are those that are still found in the results, but fall below the inclusion threshold where they had previously been included (taking into account any user intervention). Some sequences that were once significant can completely disappear from the results file, and these are considered ‘lost’. After clicking through to the results, the iteration summary box includes a link to list all ‘lost’ sequences, if appropriate. It will also include a link that allows the user to ‘Jump to the first new match’. The presence of a new match is indicated in the result table by a green background in the cell containing the sequence accession. Sequences that have been dropped will be below threshold and be indicated by a pink background in the sequence accession cell.

When running *jackhmmer* searches interactively, the user can keep iterating the search until the search converges (i.e. no new hits are found and no hits are either dropped or lost) or they deem that no further iterations are necessary. It is also possible to replicate the command line functionality of *jackhmmer* where multiple iterations are performed sequentially without intervention, by using the batch search option under the ‘advanced’ options on the search submission page. In this mode, all hits scoring above the inclusion threshold will be used in the subsequent iteration. Similarly to the command line version, the user can choose to iterate automatically for up to 5 iterations (or until convergence). As with the other searches against a target sequence database, the sequence search space can be restricted according to taxonomy, and this restriction will be applied in each successive iteration. However, if the results have been filtered according to domain architecture and/or taxonomy, all significantly scoring sequences are used in the subsequent rounds.

The interactive iterative searching is analogous to the approach adopted during Pfam curation (26) that is performed using command-line tools. The inclusion of *jackhmmer* in the HMMER site and the provision of pfamseq as a target database now provides a parallel platform to the Pfam curation system. As this system is public, it will potentially enable Pfam curation to be distributed within the scientific community.

## DISCUSSION

This article described the recent developments to the HMMER website search interface since the first release was published. Notably, all of the HMMER command-line protein search algorithms now have an equivalent that is accessible via the web. In addition to the new algorithm and additional target databases, substantial effort has been made to provide different results visualisation, which can also be used to filter the results according to taxonomy and/or domain architecture. The ability to select subdivisions of the target database (either according to predefined groups such as reference proteomes or by using taxonomic restrictions) is a complementary approach to achieving the same goal, the improved navigability of results. The HMMER development team remains committed to improving both search strategies and the presentation of results that scale well with the ever-increasing target sequence databases. However, the user interface has now reached a certain degree of stability, and what started a feasibility pilot project has now turned into a widely used informatics resource.

At the time of writing, the HMMER website is running at both Janelia Research Campus (http://hmmer.org) and the European Bioinformatics Institute (EMBL-EBI, http://www.ebi.ac.uk/Tools/hmmer). While the responsibility of the algorithm development will remain in the US, the search infrastructure and website development group have transitioned to the UK. We anticipate that the infrastructure running at Janelia Research Campus will be decommissioned during 2015. Heavy users of the API are encouraged to update their software to connect to the EMBL-EBI site. The two sites will continue to inter-operate seamlessly, with the HMMER source code and binaries being made available via hmmer.org, and the search functionality provided by the EMBL-EBI site. Both sites will adopt a common branding that is now displayed at the UK site, giving a uniform look as a user switches from one site to another.

While any change to the organisation of web services can be irksome, there are many advantages to locating the web based HMMER homology searches at EMBL-EBI. Primarily, EMBL-EBI has the infrastructure to sustain the user-base growth, while maintaining scalability of the searches against the background of ever growing sequence databases. Being co-located with the source of many of the target databases (UniProtKB, PDBe (27), Pfam) brings many benefits, as updates to the HMMER target databases will be more closely synchronised with new source database releases. Also, as the homology search system becomes established at the EMBL-EBI, there will be better cross linking to relevant databases at the EMBL-EBI and use of the search infrastructure by EMBL-EBI resources.
